# Unusual echolocation behaviour of the common sword-nosed bat *Lonchorhina aurita*: an adaptation to aerial insectivory in a phyllostomid bat?

**DOI:** 10.1098/rsos.182165

**Published:** 2019-07-31

**Authors:** Gloria Gessinger, Tania P. Gonzalez-Terrazas, Rachel A. Page, Kirsten Jung, Marco Tschapka

**Affiliations:** 1 Institute of Evolutionary Ecology and Conservation Genomics, University of Ulm, Ulm, Germany; 2 Smithsonian Tropical Research Institute, Balboa, Ancón, Panama City, Republic of Panama

**Keywords:** sensory ecology, foraging, bat echolocation, neotropics

## Abstract

Most insectivorous bat species in the Neotropical family Phyllostomidae glean insects from ground, water or vegetation surfaces. They use similar and stereotypical echolocation calls that are generally very short (less than 1–3 ms), multi-harmonic and frequency-modulated (FM). By contrast, the common sword-nosed bat, *Lonchorhina aurita*, which has the longest noseleaf in the entire phyllostomid family, produces distinctly different echolocation calls. They are composed of a constant frequency (CF) component with a peak frequency of 45 kHz, followed by a short FM down-sweep at the end. With a mean call duration of 6.6 ms (max. 8.7 ms) when flying in the open they have the longest echolocation calls reported from phyllostomid bats. In cluttered environments, the CF-component is very short. In open habitats, however, *L. aurita* can emit pure CF-calls without the terminal FM-component. We also recorded in the field a distinct terminal group that closely resembles the feeding buzzes of aerial hawking bat species from other bat families. This is the first time the echolocation call design of *L. aurita* is presented. In addition, we contrast the echolocation behaviour of individuals flying in open and confined situations. Our results suggest that the unique echolocation system of *L. aurita* represents an adaptation to aerial hawking, a very unusual hunting mode within the phyllostomid family.

## 1. Introduction

Most species of bats use echolocation to navigate and find food. Sensory constraints imposed by the foraging habitat, foraging strategy and resource type, however, determine the extent to which echolocation is used in foraging [1–3]. In acoustically complex environments such as dense vegetation, food perception by echolocation is challenging, as echoes reflected from prey insects are often masked by background echoes [4]. The New World leaf-nosed bats (Phyllostomidae) forage predominantly in dense Neotropical rainforests and are considered the ecologically most diverse bat family [5]. The dietary habits of this family range from animalivory to frugivory to nectarivory to sanguivory, with some species feeding exclusively on blood of mammals and birds [6,7]. Accordingly, the sensory systems of phyllostomids are adapted to process information from several different sensory inputs, such as the auditory channel which includes both echolocation and passive hearing (i.e. listening for sounds emitted by prey), as well as olfactory and visual channels and even thermosensation [4,8–13].

Frugivorous and nectarivorous phyllostomid bats combine sensory cues while foraging. They use olfactory cues provided by resource plants mostly for long-distance detection, while at close range they approach fruit and flowers mainly guided by echolocation [8,11,14,15]. By contrast, most of the animalivorous phyllostomids, which often show conspicuously large ears and prominent noseleaves, are considered predominantly passive gleaning foragers [16]. They rely primarily on prey-generated cues such as frog calls or katydid stridulations for prey detection, and use echolocation mostly for obstacle avoidance and general orientation [17–19]. In recent years, however, detailed studies have revealed that a few phyllostomid species have evolved specialized foraging strategies and echolocation behaviour to gain access to specific resources and habitats [1]. For example, the common big-eared bat, *Micronycteris microtis*, uses echolocation to actively glean silent and motionless prey from leaf surfaces within dense understorey vegetation [4,20,21]. The trawling phyllostomid bat, *Macrophyllum macrophyllum*, uses echolocation for the detection and localization of insect prey on and just above the water surface [22,23]. Unlike other phyllostomids, *M. macrophyllum* emits a terminal phase comparable to trawling and aerial insectivorous bats from other families, before capturing aerial prey above water [23]. Both species achieve these specific hunting tasks with small modifications of the typical phyllostomid echolocation call design, which is highly stereotypic despite the high ecological and behavioural diversity within the family. Most species emit short (1–3 ms), multi-harmonic and frequency-modulated (FM) calls at a high repetition rate. Echolocation calls may differ in peak frequency between species, but are otherwise highly conserved across the family [1,24].

**Figure 1.**
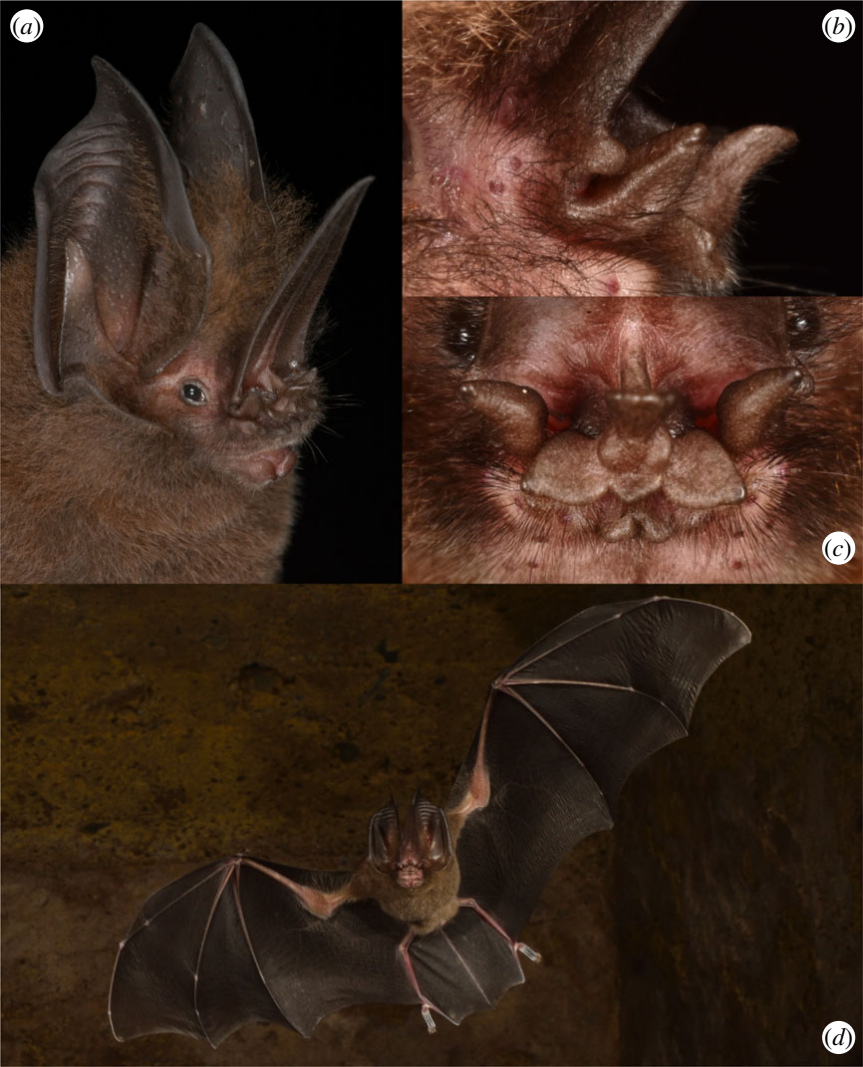
Portrait of *Lonchorhina aurita* (*a*); lateral view of basal noseleaf structures (*b*); frontal view (*c*); *L. aurita* in flight, showing the large uropatagium with tail extending to the posterior edge of the membrane (*d*).

In general, phyllostomid bats are evolutionarily highly plastic. Divergent morphologies may indicate interesting behavioural deviations from general patterns. A particularly variable morphological trait is the noseleaf, which gives name to the family. Phyllostomid noseleaves are thought to play an important role in directing echolocation calls emitted through the nostrils and vary widely in length, width and shape [25,26]. The largest and most complex noseleaves in the phyllostomid family are found in the sword-nosed bats of the genus *Lonchorhina* (figure 1*a,d*). Although little ecological information is available, *Lonchorhina* spp. are considered to be mainly insectivorous, with their large ears suggesting passive gleaning as their primary foraging strategy [27]. Within the family Phyllostomidae, the six *Lonchorhina* species (*L. inusitata, L. orinocensis, L. marinkellei, L. mankomara, L. fernandezi* and *L. aurita*) were long considered part of the subfamily Phyllostominae [28], but were recently placed into a new monogeneric subfamily Lonchorhininae [29,30]. Of all *Lonchorhina* species, the common sword-nosed bat, *Lonchorhina aurita* Tomes 1863, has the largest distribution, occurring in tropical forests throughout Central and South America. Not only have *Lonchorhina* spp. the longest noseleaf of all phyllostomids, they show also several fleshy appendages around the nostrils that are not present in other genera (figure 1*b*,*c*). In spite of the striking noseleaf morphology almost nothing is known about the foraging and echolocation behaviour of the genus, mainly because the species are not frequently captured [31].

The goal of this study was to describe and analyse the echolocation call design, variability and echolocation behaviour of the common sword-nosed bat, *L. aurita*, in Central America. We hypothesized that the striking morphology of the noseleaf should be reflected in a deviation from the general, stereotypic echolocation design and behaviour of phyllostomid species. We recorded the echolocation calls of *L. aurita* in various situations spanning a gradient of acoustic complexity, both in its natural habitat and within the confined space of a flight cage.

## 2. Methods

### 2.1. Study sites

We encountered *L. aurita* at several localities in Costa Rica and Panama in 2003 and from 2012 to 2016. In Costa Rica, we captured individuals in Tirimbina Biological Reserve, an Atlantic tropical lowland rainforest, and in the Biological Reserve Alberto Manuel Brenes, San Ramon, a tropical premontane rainforest [32]. In Panama, we captured and recorded *L. aurita* in Soberanía National Park, a semi-deciduous tropical moist forest [33]. In both countries, Costa Rica and Panama, we obtained echolocation call sequences both during free flight in rather open habitats and in confined space, thus allowing us to assess the acoustic response of the bats to different habitat structure.

### 2.2. Free-flying/relatively open space

**Figure 2.**
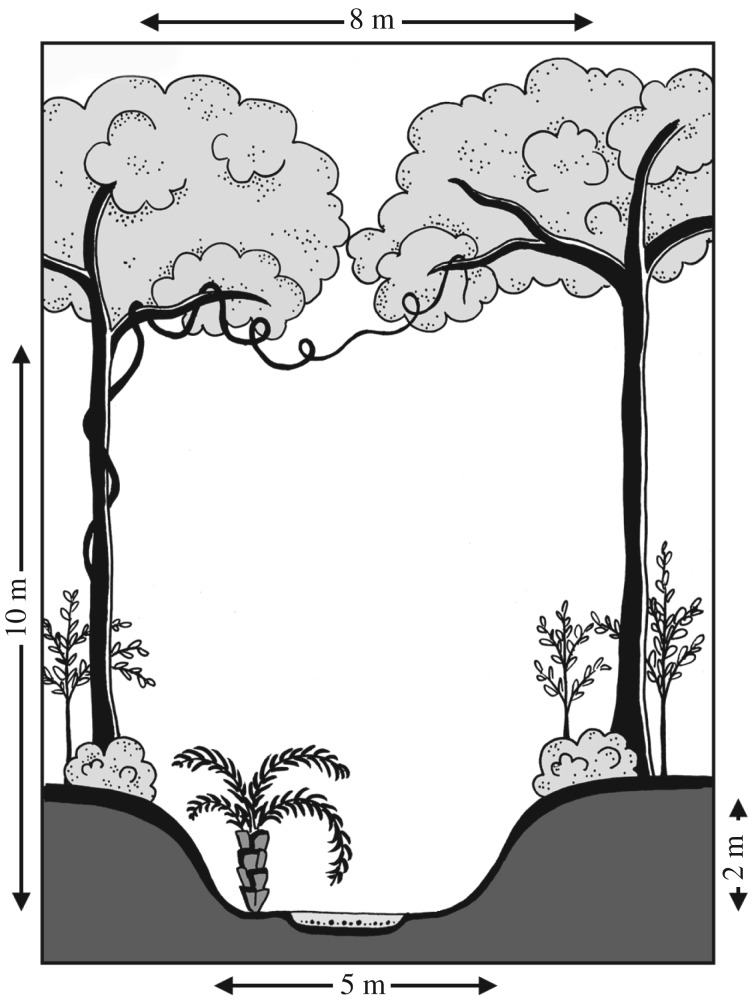
Semi-open site within the forest near Gamboa, Panama, where free-flying *Lonchorhina aurita* were recorded. The length of the gap was approximately 15 m.

Field recordings in Panama were obtained on one evening from animals free-flying in relatively open areas within the forest, near or over a small stream (figure 2). We recorded seven sequences of *L. aurita* within the first 30 min after sunset. Net captures a week prior to recording at this site in Soberanía National Park resulted in 45 *L. aurita,* all flying into the net from the same side at the beginning of the night within 30 min, so it is highly unlikely that our seven recordings of passing bats were from the same individual. In Costa Rica, we recorded one release sequence each in San Ramon and Tirimbina, respectively. Both bats were released into forest clearings and we were able to record them here for a couple of seconds free-flying before they disappeared. In San Ramon, we additionally recorded on another occasion one free-flying individual above a trail. Echolocation sequences obtained from release calls did not differ from the sequences obtained from free-flying individuals in the forest (comparison between the two release sequences and the eight free-flying sequences: call duration, *t*-test, d.f. = 8, *p* = 0.402; bandwidth, *t*-test, d.f. = 8, *p* = 0.069). Therefore, both free-flying individuals in the forest and individuals flying freely after release were merged into the single group ‘free-flying’.

### 2.3. Flight cage/confined space

The 10 sequences analysed are based on 10 different individuals captured with mist nets. In Panama, we recorded eight individuals flying in a 2.30 × 3.50 m flight cage. In Costa Rica, we recorded two individuals flying in small enclosures: one in San Ramon (*ca* 2.50 × 4.00 m) and one in Tirimbina (*ca* 3.00 × 3.00 m). Captured bats were identified, sexed and measured (weight, forearm length).

### 2.4. Echolocation recording and analyses

All echolocation sequences were recorded using CM16/CMPA microphones with the UltraSoundGate interface (Avisoft Bioacoustics, Berlin) at sampling rates of 384–500 kHz and 16 bit resolution. In total, we obtained 18 recordings of *L. aurita* in free flight and 21 in the flight cage. For analysis, we selected 20 sequences, 10 of which were from individuals passing by in natural, rather open forest situations, while 10 sequences from 10 different individuals were obtained within the confined space of a flight cage. We chose the sequences with the best signal-to-noise ratio and from these only calls with relative intensities of peak frequency above −45 dB, exceeding the background noise, were included into the analysis. Each sequence represented therefore a single individual and each contained between 10 and 26 echolocation calls.

The analysis of echolocation signals was conducted using the custom-made sound analysis software Selena (University of Tübingen, Germany) with an FFT of 512, a maximum frequency of 150 kHz, overlap of 97.87%, and a dynamic range of 60 dB (frequency resolution: 293 Hz/0.02 ms, Hamming window). We limited our analysis to the 3rd harmonic, which contained most of the sound energy. Start and end of echolocation calls were defined as −15 dB below maximum amplitude within the signal. For each sequence, we extracted temporal (pulse duration, call interval, repetition rate) and spectral parameters (peak frequency, start and end frequency). We first calculated mean values per sequence (from single individuals) and then combined these individual-based values for calculating the overall means for the respective group.

For determining bandwidth of the constant frequency (CF-) component, we measured peak frequency at 10 selected CF-calls (from 10 individuals). In order to exclude the initial and final FM-sweep, we discarded the first and the last quarter of each call and, based on the full temporal resolution, measured bandwidth only of the central section.

To test whether the presence of acoustic clutter in the habitat influenced echolocation call parameters in *L. aurita*, we compared echolocation call parameters per sequences obtained in relatively open habitat and sequences obtained in confined space using *t*-tests with a critical alpha of 0.05.

## 3. Results

### 3.1. Echolocation call design

**Figure 3.**
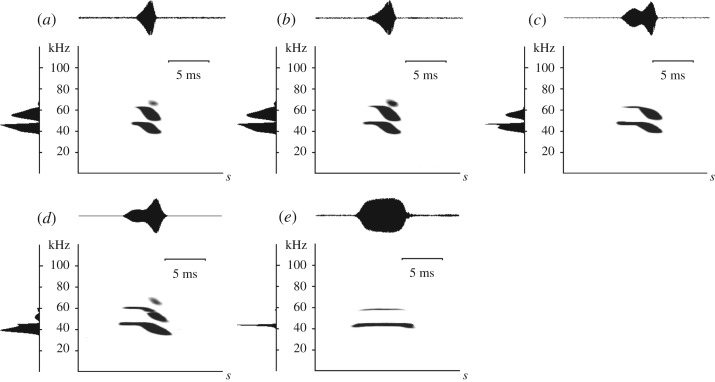
Search call repertoire of *L. aurita,* arranged from confined and cluttered (*a*,*b*) to more open recording situations (*c*–*e*) such as in figure 2. Each diagram provides spectrogram with normalized oscillogram (above) and power spectrum (left). Spectrogram parameters: FFT 1024, Hann window 96% overlap.

Echolocation calls of *L. aurita* are composed of an initial CF-component with a peak frequency at 45 kHz, and a terminal FM-component (figure 3). Mean bandwidth of the CF-component was 587 Hz, which corresponds to twice our frequency resolution, indicating that the bats strictly maintained a CF without any modulation (electronic supplementary material, table S1). Echolocation calls, however, revealed a high variability in call structure with the duration of the CF-component changing systematically with habitat clutter, being extremely short and barely visible in some calls (figure 3*a*,*b*) to representing *ca* 50% of pulse duration of the total call (figure 3*c*,*d*). We also regularly recorded pure CF-calls of extremely narrow bandwidth and without a distinct terminal FM part (figure 3*e*). Echolocation calls also varied extremely in overall call duration, ranging from calls with only 1.1 ms to calls with 8.7 ms duration. While in the shorter calls, sound energy was distributed almost equally between the 3rd and 4th harmonic (figure 3*a–c*), in longer calls the energy was concentrated in the 3rd harmonic (figure 3*d*,*e*), with the 4th harmonic disappearing almost completely (figure 3*e*; table 1).

**Table 1.** Means (±s.d.) of basic call parameters of *Lonchorhina aurita* flying in a confined space (flight cage, *n* = 10 sequences from 10 bats) and in relatively open areas (free-flying in forest gaps, *n* = 10 sequences, compare figure 2). Values are means of individual-based means.

	flight cage	free-flying			
	mean ± s.d.	mean ± s.d.	*t*-test	d.f.	*p*-value
peak frequency (kHz)	44.59 ± 1.02	45.67 ± 1.99	*t* = −1.540	18	0.141
bandwidth (kHz)	9.05 ± 0.88	7.54 ± 1.15	*t* = 3.298	18	0.004*
pulse duration (ms)	2.77 ± 0.61	6.55 ± 0.67	*t* = 13.20	18	<0.001**

### 3.2. Contrasting calling behaviour in flight cage and in the field

**Figure 4.**
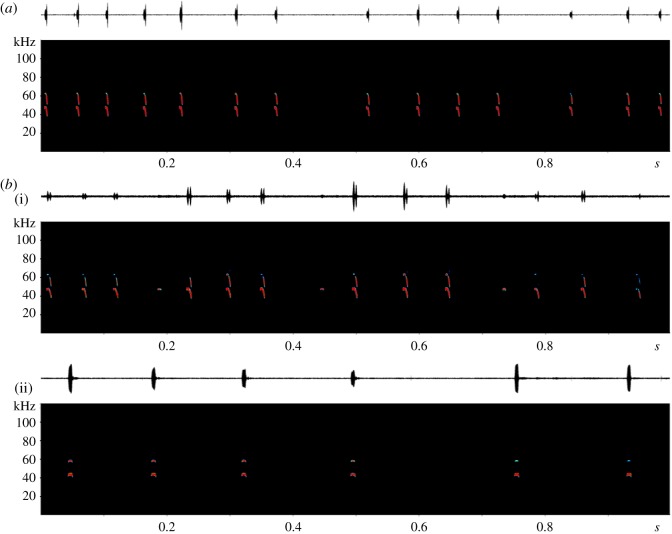
Complete sequences of *L. aurita* flying inside a flight cage (*a*) and during free-flying flights in a semi-open area (*b*i) and in an open area (*b*ii). Spectrogram parameters: FFT 1024, Hann window, 83% overlap.

Although we did not measure actual call intensity, the vocalizations of *Lonchorhina aurita* in the flight cage appeared to be of similar intensity to those of other similar-sized phyllostomids, such as *Carollia perspicillata,* recorded in the same situation*.* Within the confined situation of a flight cage, we observed orientation calls of *L. aurita* with very short CF- and more prominent FM-components, with grouping of calls at intermediate repetition rates and pulse intervals around 50 ms within the groups (figure 4*a*). Only one sequence from the flight cage contained pure CF-calls (total number of calls: 19, number of CF-calls: 2, percentage of pure CF-calls: 10.5%; electronic supplementary material, table S2).

By contrast, when we recorded free-flying *L. aurita* in relatively open situations in the forest, i.e. below the canopy over streams, in tree-fall gaps or above trails and small roads (figure 2), echolocation calls were characterized by a prominent CF-component followed by an FM-sweep (total number of calls: 270, number of CF-calls: 102, percentage of pure CF-calls: 37.8%, electronic supplementary material, table S2). We also observed grouping of calls in the free-flying bats; however, in this case, an initial pure CF-call was followed by several CF-FM-calls (figure 4*b*i). In some cases, FM-components disappeared almost entirely, resulting in a sequence of only CF-calls with extended pulse intervals of more than 150 ms (figure 4*b*ii).

While we found no significant difference in peak frequency between flight cage and field situations, call duration recorded in the field was more than double as long and thus significantly higher than in the flight cage (*t*-test, d.f. = 18, *p* < 0.001). Echolocation call bandwidth, however, was significantly higher in the flight cage than in the field (*t*-test, d.f. = 18, *p* < 0.01; table 1; electronic supplementary material, table S3).

### 3.3. Hunting behaviour

**Figure 5.**
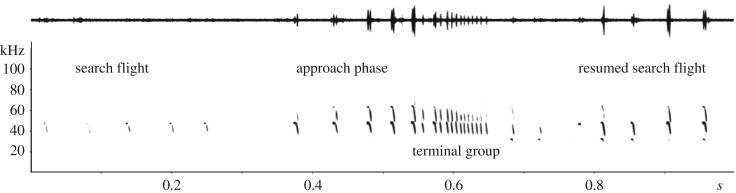
Terminal group of *L. aurita* recorded in a semi-open site near a stream (figure 2). Spectrogram parameters: FFT 1024, Hann window 83% overlap.

In the field, we obtained one recording of a free-flying *L. aurita* emitting a terminal group (figure 5), before returning to search call emission. The sequence started with the signature CF-FM-calls only found in *Lonchorhina* and proceeded to a series of FM-calls with a minimum call duration of 1.3 ms and minimum pulse interval of 5.1 ms (number of calls in entire sequence: 28, number of calls in terminal group: 12).

## 4. Discussion

Bat echolocation call structure ranges from FM-calls covering a high bandwidth, to quasi-constant calls (QCF) with low-frequency bandwidth, to CF-calls where essentially the entire call energy is concentrated within an extremely narrow frequency range [16,34]. Different call structures provide different functional context. While FM-calls allow a precise measurement of distance and location of targets even in complex habitats due to a precise time–frequency relationship, QCF or CF-calls increase detectability of low intensity echoes over larger ranges, e.g. from airborne prey in more open habitats [35–38]. The comparably few bat taxa using long CF-calls are highly specialized to detect insect wingbeats close to background vegetation (for details, see [16,34]). While some characteristics of echolocation remain rather constant within a species and thus allow a general categorization into one of these three main echolocation types, bats modify essential echolocation parameters continuously to the specific sensory situation, particularly in the temporal domain such as call length, pulse interval and sweep rate.

Phyllostomid echolocation consists mainly of short (1–3 ms), multi-harmonic and steeply FM-calls. These highly stereotypical calls are considered an adaptation to close-range orientation within a highly complex (cluttered) environment such as the forest understorey [24]. The structure of echolocation calls within the entire phyllostomid family is remarkably uniform, which makes identifying bats of this family by echolocation calls alone nearly impossible [24]. *Lonchorhina aurita* deviates in its echolocation call structure conspicuously from the standard phyllostomid design with a unique initial CF-component of variable length, followed by an FM-component, although the latter may also be lacking. These unusual vocalizations, particularly the CF-calls (figures 3*e* and 4*b*ii), are not always readily recognizable as phyllostomid signals. However, as in most phyllostomids, the main energy of *L. aurita*'s echolocation calls lies in the 3rd harmonic with the 4th harmonic often present, while the fundamental and 2nd harmonic are suppressed [24].

Recent evidence demonstrates that some phyllostomid species use significantly longer echolocation calls in open space as compared with the cluttered environment inside the forest and even deviate from the standard phyllostomid echolocation call pattern. While the trawling *Macrophyllum macrophyllum* has a mean call duration of 3.6 ms and uses standard phyllostomid calls in the open (clutter: 1.3 ms) [39], the nectar-feeding *Phyllonycteris poeyi* uses 4.7 ms long vespertilionid-like echolocation calls (clutter: 2.4 ms) [40]. Similarly, the vampire bat, *Desmodus rotundus*, was recently reported to use 5.5 ms long calls with an initial QCF-element when flying in the open (clutter 1.1–2.3) [41,42]. To our knowledge, *L. aurita* has the longest echolocation calls described for phyllostomid bats with a mean duration of 6.6 ms when flying in relatively open spaces within the forest.

We found that the echolocation call design of *L. aurita* is highly variable and that calls change drastically in response to the respective environment. Within the confined space of a flight cage, *L. aurita* presented the short multi-harmonic FM-calls of phyllostomids [24,43], however, usually with the unique addition of a short initial CF-component. In relatively open space, *L. aurita* increased the duration of this CF-component, reduced the bandwidth of the echolocation call, and emitted sequences of long CF-calls with increased inter-pulse intervals. An analogous reduction of bandwidth correlating with the movement from edge space to more open areas was found for the edge foraging aerial insectivore *Myotis nigricans* (Vespertilionidae) [44]. Similarly, the vespertilionid *Vespertilio murinus* uses long echolocation calls in the open (11.2 ms) but shortens its call duration (7.1 ms) and increases bandwidth when approaching closer than 5–6 m to the background, indicating this distance as its perceived threshold between edge and open space [45]. Spaces of even larger dimensions are available within the rainforest habitat of *L. aurita* in the hall-like structures below the canopy (figure 2). We thus argue that the use of long CF-calls in these habitat types indicates that this species, coming from a family generally adapted to highly cluttered habitats, perceived such spaces under the forest canopy as relatively open. The echolocation design of *L. aurita* might reflect a unique evolution towards an unusual foraging strategy for phyllostomids. Shallowly modulated, long echolocation signals are generally seen as an adaptation to detecting faint echoes, and enable a bat to recognize airborne prey targets over larger distances [34]. Structurally similar calls are used at the study site by edge space aerial insectivores such as the emballonurids *Saccopteryx bilineata* or *Centronycteris centralis* [46].

We also recorded a distinct terminal group of *L. aurita* flying in the natural habitat (figures 2 and 5). Similar behaviour was previously only reported from two other phyllostomids, the trawling *Macrophyllum macrophyllum* [47] and the nectar-feeding *Leptonycteris yerbabuenae* [48]. The terminal groups of *L. aurita* resemble most closely feeding buzzes of aerial insectivore bats from other families when catching airborne prey [49,50]. Feeding buzzes aid in maximizing the retrieval rate of spatial information needed for flight coordination immediately before a capture attempt, particularly of moving prey objects, such as flying insects. Both the conspicuous echolocation call structure as well as the pronounced terminal group point towards the capture of aerial prey as a main hunting strategy of *L. aurita*.

Most aerial insectivores have an uropatagium stabilized by tail vertebrae that is used for handling prey immediately after capture. This feature is highly unusual within the entire Phyllostomidae family. However, all *Lonchorhina* species show a long tail that extends to the end of the large uropatagium (figure 1*d*) [51]. Interestingly, the only other phyllostomid with such a long tail is *Macrophyllum macrophyllum*, which also produces terminal groups and mainly trawls insects from water surfaces but has been shown to also capture flying insects [23,52].

The intricate noseleaf morphology of *L. aurita* (figure 1) suggests a nasal call emission. The highly complex structures around the nostrils in *Lonchorhina* spp. bear similarities to the nasal structures of the nose-emitting Old World Hipposideridae and Rhinolophidae [53]. This resemblance may indicate specialized echolocation emission through the nostrils in *Lonchorhina*. Interestingly, *Lonchorhina* calls also resemble the echolocation signals of the nose-emitting Hipposideridae. Hipposiderids frequently combine short CF- with FM-components (e.g. *Cloeotis percivali, Hipposiderus caffer* [54]; *Hipposiderus larvatus* [55])*.* Although Hipposideridae in general show much shorter CF-components than Rhinolophidae, they are also able to detect glints from the wingbeat of fluttering targets, i.e. flying insects. A trained *Hipposideros speoris* was able to discriminate between fluttering and motionless targets using CF-components of *ca* 5 ms length [56]. Considering an insect with a 50 Hz wingbeat, i.e. one wingbeat every 20 ms, each of the 7 ms long CF-calls from *L. aurita* would have a 35% probability of producing an echo containing a glint.

The unique echolocation call design of *Lonchorhina* spp. might allow the inclusion of this phyllostomid genus in acoustic monitoring efforts, as suggested for *Lonchorhina inusitata,* the only other published species [57]. The highest energy in the 3rd harmonic is a clear giveaway for a phyllostomid call; however, calls of *L. aurita* are not overly loud. In lower-quality and off-axis recordings, with missing harmonics, calls of *L. aurita* could potentially be confused with emballonurid species, such as *Centronycteris centralis*, that hunts in similar habitat and shows a QCF-peak frequency of 41.3 kHz in the 2nd harmonic [46]. Therefore, high-quality recordings with harmonics are critical for reliable differentiation between *L. aurita* and other edge space bats.

In conclusion, we found that the unusual morphology of the noseleaf is indeed reflected in a distinct deviation from the general, stereotypic phyllostomid echolocation design and behaviour, and thus were able to confirm our initial hypothesis. The combination of long CF-calls, the terminal group and the morphology of the tail membrane suggest that *L. aurita* employs a predominantly aerial insectivore hunting strategy in the relatively open space below the forest canopy. As such, *L. aurita* expands the phylogenetic heritage of the phyllostomid family towards the niches of aerial insectivore families such as Emballonuridae and Vespertilionidae. The echolocation system of *L. aurita* retains basic phyllostomid characteristics such as multi-harmonic FM-calls, but, as is typical in aerial insectivores, it has evolved to also include narrow-bandwidth CF-components and a terminal group. Further study in the field as well as experiments in the laboratory are needed to understand the functional significance of *L. aurita*'s unique noseleaf, and to determine whether *L. aurita* is in fact the first glint-detecting phyllostomid bat. A detailed analysis of prey species consumed will offer critical information on the hunting behaviour associated with the unusual echolocation of this enigmatic species.

## Supplementary Material

Bandwidth of the constant frequency component (CF)

## Supplementary Material

Percentage of CF and CF-FM calls.

## Supplementary Material

Echolocation parameters of Lonchorhina aurita

## Data accessibility

The dataset supporting this article has been uploaded as part of the electronic supplementary material.
